# Suicides in the COVID-19 Pandemic — Are We Well Informed Regarding Current Risks and Future Prospects?

**DOI:** 10.17816/CP56

**Published:** 2021-03-20

**Authors:** Vsevolod A. Rozanov, Natalia V. Semenova, Aleksandr Ja. Vuks, Victoria V. Freize, Vladimir D. Isakov, Orazmurad D. Yagmurov, Nikolay G. Neznanov

**Affiliations:** St. Petersburg State University; V.M. Bekhterev National Medical Research Center for Psychiatry and Neurology; St. Petersburg Bureau of Forensic Medical Examinations; I.P. Pavlov First St. Petersburg State Medical University

**Keywords:** suicide, COVID-19, pandemic, crisis, acute phase, containment measures, самоубийства, COVID-19, пандемия, кризис, острая фаза, самоизоляция

## Abstract

**Background.:**

Suicides are predicted to drop in the acute phase of any crisis that poses a threat to the entire population, though data on this are inconsistent. A pandemic is the most severe global crisis one can imagine. There is an urgent need to identify objective trends in suicide rates across countries and populations in a real-time manner in order to be better informed regarding prospects and adaptation of preventive strategies.

**Objectives.:**

To evaluate suicidal behaviour in a metropolis immediately after the introduction of severe containment measures due to the pandemic.

**Methods.:**

Cases of completed suicides in St. Petersburg were obtained from the local city Bureau of Forensic Medical Examinations for the period 1 January 2016 to 31 July 2020. Data were accurately collected and monthly frequencies per 100,000 of the population in April-May 2020 (introduction of the most severe stay at home measures) were compared with corresponding data from 2016-2019. Confidence intervals were calculated according to Wilson.

**Results.:**

Suicide frequencies in the population of St. Petersburg in April 2020 did not go up, in contrast, they were 30.3% lower than the average for the previous four years. The decrease in April was more pronounced in males than in females (36.3% and12.4%, respectively). When looking at age groups it was found that the biggest drop in suicides was in older males ( 55 years). In this group, suicide indices were 58.5% lower than average for the previous four years. However, in females, there was a 50% rise in suicides in June, while in young males (15-34 years) there was an 87.9% rise in May. Total number of suicides for the first half of 2020 was very close to the average seen in previous years. None of the registered changes were statistically significant.

**Conclusions.:**

The analysis is preliminary and does not account for possible seasonality, however, we consider that the reduction in completed suicides immediately after crisis exposure deserves attention. It supports views that in the acute phase of the crisis, suicidal behaviour may decline, which may be quickly replaced by a rise. Such a rise in females and younger males points on possible risk groups and requires a response from society. More studies are needed to have a clearer picture of suicide dynamics in Russia during the different waves of the pandemic, and prevention should be prioritized regardless of the tendencies.

## INTRODUCTION

The COVID-19 pandemic has dramatically changed the life of billions of people all around the world. Among the many anticipated negative health effects, there has been concern from psychiatrists and psychologists about the increasing risk of suicide [1,2]. Some of the authors have even used the theory of a “perfect storm” for modelling possible consequences of a pandemic for suicidal behaviour [3]. There were concerns expressed that risk may increase due to the anxiety, depression and sleep disturbances during social isolation, economic stress and unemployment, fear of catching the disease, distress due to family members suffering from the disease, stigmatization of patients with COVID-19 and their families, as well as alcohol consumption and domestic conflict during quarantine [4,5]. Quickly-organized studies (mostly internet surveys) have revealed that feelings of being stressed, depressive symptoms and anxiety have increased during the harshest containment measures, especially in students, women, and medical staff [6-8]. Negative expectations were expressed widely, however objective studies are essential to evaluate the suicide risk in any specific crisis, social situation, or disaster, with regard to most vulnerable demographic, social and occupational groups.

Humanity has experienced several severe epidemics that may serve as quasi-experimental situations relevant in this sense. Therefore, the question is what can be learned regarding changes in suicide rates from previous cases? Quite surprisingly, the information appears to be limited and inconsistent. In the 1990s, American suicidologist Ira Wasserman, used official statistics to assess the impact on suicide deaths of three major social events in the USA in the period from 1910 to 1920: World War I (1914-1918), the Spanish flu pandemic (1918-1920) and the introduction of the “prohibition law" (since 1919). According to his analysis, the war did not have any effect, the pandemic led to an increase in rates of suicide, while alcohol restrictions led to a decrease [9]. Later, in a study from Hong Kong, where the SARS epidemic had caused high mortality between 1993 and 2004, authors have found a significant increase in suicide rates among older people (>65 years) once the peak of the epidemic had passed [10].

This scanty list gives the impression that previous pandemics (or more local epidemics) did not attract much attention from suicidologists. However, attention was paid to other types of crises that confronted humanity across history, such as wars, natural catastrophes, and other disasters. Emile Durkheim, in his sociological study, has pointed out that such events usually lead to a lowering of suicide rates [11]. He discussed this from the point of view of the “pulling together” effect in society that unites people in the face of a critical threat. In support of this, several reports from different countries shortly after the announcement of the COVID-19 pandemic, have presented data that show that the number of suicide attempts and suicides did not increase, on the contrary, they seemed to go down during the introduction of “stay at home” orders [12-14].

While this immediate effect may be understood from the point of view of sociological theory, further tendencies during a pandemic need much more attention and objective description. As a response to this challenge, an international initiative, COVID-19 Suicide Prevention Research Collaboration, was recently established. The initiative aims to monitor suicide rates and develop adequate prevention measures [15]. The initiative that now unites more than 30 countries, aims to provide a thorough analysis of the situation in a real-time manner. This implies better interdisciplinary interaction and communication, involving sociologists, psychologists, psychiatrists, forensic medicine specialists, and the law enforcement system. Here we provide our experience in establishing and developing such communication, which eventually resulted in some preliminary observations regarding the immediate change in suicide rates in St. Petersburg shortly after the introduction of the strict quarantine on 30 March.

## MATERIAL AND METHODS

In the middle of April, the Ministry of Public Health of the Russian Federation issued a letter to the leading centres of psychiatric research requesting that they evaluate the possible evolution of risk for completed and attempted suicide in the pandemic. As a response to this request, V.M. Bekhterev National Medical Center of Psychiatry and Neurology, in collaboration with scientific and educational medical institutions in St. Petersburg, made an effort to collect relevant data. Our primary aim was to evaluate the possible changes in suicidal behaviour during the earliest phase of the crisis in St. Petersburg with the prospect of prolonging the observations and widening the catchment area. Numbers and demographic data of those who died by suicide from 1 January 2016 (first available year) to 31 July 2020 (the last point for confirmed suicides, previously referred to as “probable”) were obtained from the St. Petersburg Bureau of Forensic Medical Examinations.

The initial data (suicide cases per month in absolute units) were recalculated as monthly frequencies per 100,000 of the population for the period 2016-2019 and were compared with corresponding months in 2020. The methods of Fisher, Clopper-Pearson, and Wilson were tested to calculate the confidence intervals (CI). Finally Wilson’s method was selected. Demographic data on the population of St. Petersburg for the specified period were obtained from official sources (Rosstat).

## RESULTS

**Figure 1:**
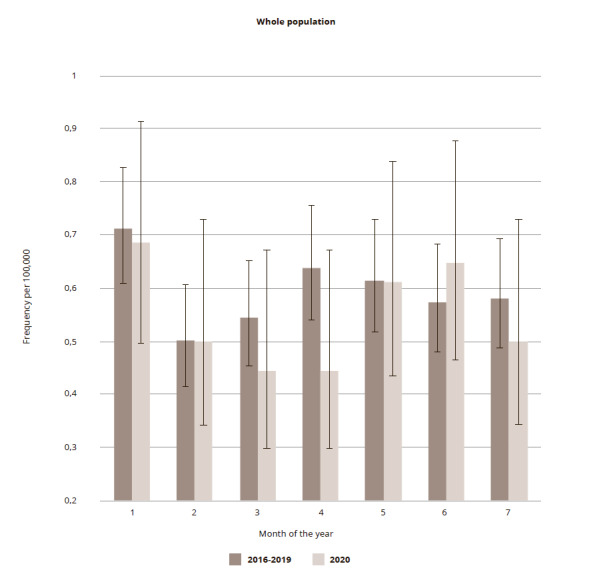
Figure 1. Suicide frequencies in St. Petersburg per 100,000 of the whole population in the first 7 months of the year 2020 in comparison to corresponding period average in 2016-2019

In the current study, a set of suicide cases (n= 1.647) that occurred in St. Petersburg in the period from 1 January 2016 to 31 July 2020 is used. Of these cases, 427 were female and 1,220 male (M : F ratio = 2.857). Completed suicides for the whole population in the first seven months in 2016-2019 and separately in 2020 are presented in Figure 1. A decrease in the frequency of suicides per month per 100,000 population can already be seen in March 2020. The frequency decreased by 18.3% (0.4446, 95% CI: 0.2988 – 0.6616 as compared with 0.5440, 95% CI: 0.4536 – 0.6525) with a more pronounced decrease in April of 30.3% (0.4446, 95% CI: 0.2988 – 0.6616) as compared with 0.6378 (95% CI: 0.5393 – 0.7544). However, this decline was not statistically significant. Subsequently, there was an increase of 13.3% in June and then a decrease of 14.0% in July. The total accumulated number of completed suicides in the first six months of 2020 constituted 93.8% of the average in the first six months in 2016-2019.

**Figure 2:**
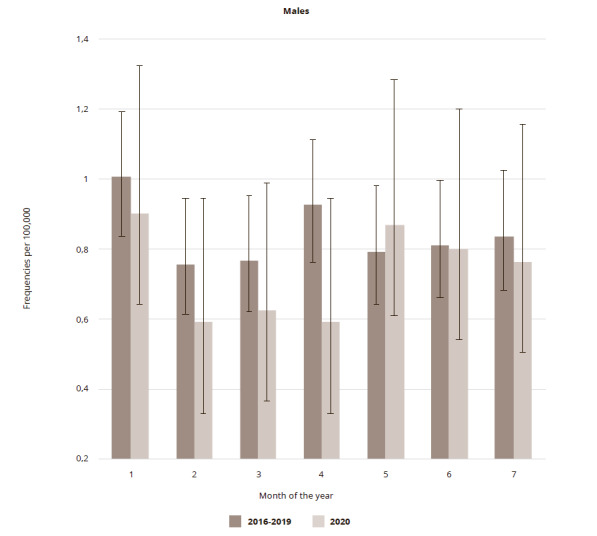
Figure 2. Suicide frequencies in St. Petersburg per 100,000 male population in the first 7 months in 2020 in comparison to corresponding period average in 2016-2019

**Figure 3:**
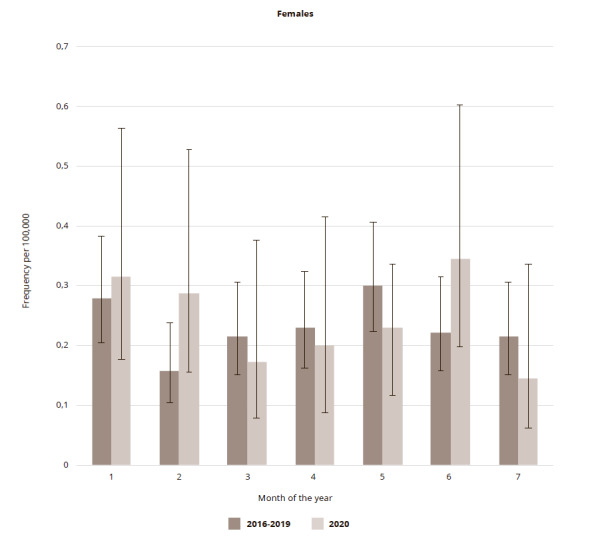
Figure 3. Suicide frequency in St. Petersburg per 100,000 female population in the first 7 months in 2020 in comparison to corresponding period average in 2016-2019

A closer look revealed that the decline in March and April 2020 was more pronounced among men (18.4% and 36.3%, respectively). For men, at the lowest point (April 2020) the frequency per 100,000 reached 0.5898 (95% CI: 0.3683 – 0.9446), though the fall was also insignificant. Among women, the decrease in the frequency of suicides per month in 2020 was 20.2, 12.4, and 23.8% in March, April, and May, respectively (the lowest level in May was 0.2293 (95% CI: 0.1161 – 0.4524). All changes were insignificant (Figures 2‒3). The total number of male cases in the first six months of 2020 was 88.7% of the average for the same period in 2016-2019, whilst among women it increased by 10.2%.

It can be seen that after a short decline in the suicide frequency for males, the trend reversed in May, whilst for females, there was a 50% rise in June. None of the observed fluctuations in rates were significant. Since the most pronounced changes were seen in the male population, the fluctuations in the frequency of suicides in different age groups of men were scrutinized. Given the comparatively small number of cases per month, the following wide age groups were chosen: young (15-34), mature (35-54), adults (55-74), and seniors (>75). It was found that from January to March, among men of different age groups, the suicide rate changed direction, while in April a decrease was observed in all groups, with the most pronounced (58.3 and 58.7%) among men aged 55-74 years, and 75 years and over, respectively. By May, this decline gave way to a rise (87.9%), which was most pronounced among young men aged 15-34.

## DISCUSSION

There is almost complete agreement in suicidology that in the acute phase of any crisis (war, terrorist violence, natural disaster, or mass infection) suicides usually go down [16]. The recent coronavirus crisis in this sense is a “perfect storm” – it is global rather than local, like a tsunami or an earthquake, and more dangerous than war conflict due to the inability to escape or identify the source of danger [17]. Since Durkheim, the main explanation for this effect is that the number of suicides goes down due to social integration and strengthening of the invisible links that make societies more united in the face of a danger to the whole population [11].

Our results are consistent with these studies that find or predict a drop in suicide incidence in the acute phase of a crisis [12-14]. We have used a blunt method of evaluation of suicide incidence change known as excess mortality – comparison between incidence during the fixed period (i.e., April) in four to five previous years and in the index year, and covering adjusted periods. April is the best period from this point of view, since government containment measures had just been introduced, and they were rather harsh and severe (described by some authors as Draconian) [18]. This can provide further explanations for the drop in the number of suicides. In this sense, it is necessary to mention that not only did the number of completed suicides fall, but also the number of attempted suicides, hospitalizations and referrals to mental health providers, as well as psychiatric emergency consultations [19]. This is confirmed by observations from St. Petersburg ambulance service, which registered a substantial drop in the number of self-intoxications in April, and by specialists in the clinical departments at the V.M. Bekhterev Center. Therefore, not only actualization of vital (adaptive) tendencies and societal cohesion, but also a decline in psychological (and even psychiatric) problems, may contribute to the observed tendency [20].

Our evaluations have several limitations, including the inability to encounter and eliminate fluctuating seasonal peaks and falls and the influence of the general trend (over the last seven to ten years, suicide rates in Russia have been slowly, but consistently going down). However, we consider that our findings deserve some attention. The objective studies regarding suicide rates during a pandemic are still scarce and every piece of knowledge may be important. In each country, given the unique political, economic, social, and cultural situation, the change in suicide rates may differ. Moreover, within professional circles and the general public, our results may serve the goal of raising awareness regarding suicide.

These preliminary results should not become a reason for complacency and denial of a possible increase in suicides in the future. We would like to draw special attention to the quick reversal to a decrease in suicide rates in May in young males and in June in females. With the complete results from 2020, a clearer picture will appear, however, it is already necessary to develop more efficient (or adjust existing) preventive measures with an eye to future periods of the development of the pandemic situation. This is important in view of the possible accumulation of economic problems within families (in spite of all the compensatory measures taken by the government), such as rising unemployment, the bankruptcy of small businesses, as well as academic stress affecting young people who seem to be less resilient to global shocks and who have found themselves in a dramatically changed educational environment with online education.

The pandemic is not over, and new emerging waves are rather unpredictable. Several studies from other sites have already registered a disturbing evolution of suicides, for instance, in Japan after a reduction in suicide numbers (14%) in the first six months of the pandemic (February to June 2020), the monthly suicide rate increased by 16% during the second wave (July to October 2020), with a larger increase among females (37%) and children and adolescents (49%) [21].Some researchers are returning to records of mortality during the Spanish flu pandemic 100 years ago. For instance, in a study from Taiwan it was shown that during the first wave, when about 22% of the population were infected, suicide rates were no higher than expected, while in the second wave at a time when only 4.3% were infected, there was an increase in suicide indexes (33-35%) at the beginning of this wave [22].

In a pandemic, new efforts are needed to organize and implement suicidal prevention measures. We fully agree with the statement that while we are waiting for a clearer picture, prevention measures must be prioritized [23]. Existing evidence-based studies provide a set of relevant strategies that require careful adaptation and tuning to be implemented in a pandemic situation. In Russia, along with the use of all the accumulated world experience, it would be reasonable to pay more attention to educational technologies, raising the status of suicidology as an academic discipline and generalizing the existing regional experience in suicide prevention.

## CONCLUSIONS

We are providing preliminary evidence that during the period of most severe restrictions due to the COVID-19 pandemic in a metropolis of 5.5 million people in the north-western region of Russia, suicidal behaviour did not increase, on the contrary, it seems to have fallen. This observation is consistent with the point of view that during the acute phase of a crisis, the number of suicides usually goes down. However, we are still not sufficiently informed regarding the situation with suicides across the huge territories and diverse ethnic and cultural groups of the Russian Federation. Further monitoring and data accumulation from wider populations and federal entities are needed to draw more informed conclusions regarding the impact of COVID-19 on suicide rates in Russia, with special attention to specific age groups. The decrease may turn into an increase when long-lasting effects accumulate, therefore even more effort is needed to enhance prevention activities.
